# Revolutionizing cancer treatment: nanotechnology-enabled photodynamic therapy and immunotherapy with advanced photosensitizers

**DOI:** 10.3389/fimmu.2023.1219785

**Published:** 2023-10-04

**Authors:** Jiedong Jia, Xue Wu, Gongwei Long, Jie Yu, Wei He, Huiping Zhang, Dongwen Wang, Zhangqun Ye, Jun Tian

**Affiliations:** ^1^ Department of Urology, National Cancer Center/National Clinical Research Center for Cancer/Cancer Hospital Shenzhen Hospital, Chinese Academy of Medical Sciences and Peking Union Medical College, Shenzhen, China; ^2^ Department of Medical Oncology, National Cancer Center/National Clinical Research Center for Cancer/Cancer Hospital Shenzhen Hospital, Chinese Academy of Medical Sciences and Peking Union Medical College, Shenzhen, China; ^3^ School of Life Science and Technology, Wuhan Polytechnic University, Wuhan, China; ^4^ Department of Urology, Tongji Hospital, Tongji Medical College, Huazhong University of Science and Technology, Wuhan, China; ^5^ Institute of Reproduction Health Research, Tongji Medical College, Huazhong University of Science and Technology, Wuhan, China; ^6^ Department of Urology, National Cancer Center, Cancer Hospital, Chinese Academy of Medical Sciences and Peking Union Medical, Beijing, China

**Keywords:** nanotechnology, photodynamic therapy, immunotherapy, photosensitizers, cancer treatment, photosensitizer

## Abstract

Nanotechnology-enhanced photodynamic therapy (PDT) and immunotherapy are emerging as exciting cancer therapeutic methods with significant potential for improving patient outcomes. By combining these approaches, synergistic effects have been observed in preclinical studies, resulting in enhanced immune responses to cancer and the capacity to conquer the immunosuppressive tumor microenvironment (TME). Despite challenges such as addressing treatment limitations and developing personalized cancer treatment strategies, the integration of nanotechnology-enabled PDT and immunotherapy, along with advanced photosensitizers (PSs), represents an exciting new avenue in cancer treatment. Continued research, development, and collaboration among researchers, clinicians, and regulatory agencies are crucial for further advancements and the successful implementation of these promising therapies, ultimately benefiting cancer patients worldwide.

## 1 Introduction

In 2020, there will be approximately 19.3 million new cases of cancer and 10 million cancer-related deaths globally, making cancer the primary cause of death (1). Surgical, chemotherapeutic, and radiotherapeutic interventions are the traditional cancer treatment approaches. Although significant advancements have been made in cancer treatment, these conventional methods frequently result in serious side effects, limited therapeutic efficacy, and the development of drug resistance (2). Consequently, there is an ongoing need for innovative and effective cancer treatment approaches.

PDT is a minimally invasive cancer treatment that uses a PS, a particular wavelength of light, and oxygen to generate reactive oxygen compounds that can cause tumor cell death (3). Upon absorbing a photon, a PS transitions from its ground state (S0) to an excited singlet state (S1 or S2). The S2 state quickly decays to S1 via internal conversion, and the unstable S1 either emits light (fluorescence) or generates heat, or may even undergo intersystem crossing to a more stable triplet state (T1). T1, with a longer lifetime, can lead to phosphorescent emission and energy transfer to O_2_ to generate singlet oxygen (^1^O_2_) – a process referred to as type II PDT. Alternatively, T1 can react with intracellular substrates to form radicals that further react with H_2_O or O_2_ to produce other reactive oxygen species (ROS) like hydroxyl and superoxide radicals - known as the type I PDT process. In most cases, the predominant photosensitizing mechanism involves the formation of ^1^O_2_, causing PDT destruction to biological tissues and cells (4).

PDT has demonstrated promising results in the treatment of different malignancies, including superficial bladder cancer, early and obstructive lung cancer, Barrett’s esophagus, head and neck cancers, and skin cancer (4, 5). Immunotherapy, on the other hand, aims to harness the immune system of patients to combat cancer by activating the immune system’s cells to recognize and eliminate cancer cells (6). Several immunotherapy approaches, including immune checkpoint inhibitors, cancer vaccines, and adoptive T cell therapy, have demonstrated remarkable effectiveness in the treatment of malignancies like melanoma and non-small cell lung cancer (7, 8). Although immune checkpoint therapy offers numerous advantages, a considerable proportion of patients with different cancer types do not respond effectively to immune checkpoint inhibitors (9). This lack of sensitivity can be attributed to their tumors’ low immunogenicity, which limits the therapeutic potential of this approach for these particular patients (10, 11).

Recent studies have highlighted the potential synergistic effects of combining PDT and immunotherapy (12). PDT can induce immunogenic cell death (ICD), which promotes the release of tumor antigens and enhances the activation of antigen-presenting cells (13, 14). This process can stimulate a robust immune response and improve the efficacy of immunotherapy (15, 16). Furthermore, combining PDT and immunotherapy can overcome the limitations of each individual treatment modality, such as the hypoxic TME in PDT and the low response rates of some patients to immunotherapy (17).

Nanotechnology is now recognized as a promising instrument for improving the delivery, efficacy, and safety of cancer therapeutics, including PSs and immunotherapeutic agents (18). The integration of PDT and immunotherapy has resulted in promising advances in cancer treatment (19–21). The use of nanotechnology to improve the safety and effectiveness of PSs and immunotherapeutic agents has opened new avenues for further exploration. This article provides a thorough analysis of the applications of nanomedicine in PDT and immunotherapy. In addition, this article also discusses the advantages of nanomedicines as PSs. Finally, the challenges and prospective future developments in the field of nanomedicine-driven photodynamic tumor immunotherapy are highlighted.

## 2 PSs for PDT

### 2.1 First-generation PSs

The pioneering PSs, known as first-generation PSs, largely comprise hematoporphyrin derivative (HpD) and Photofrin. Hematoporphyrin was one of the first agents discovered to have photosensitizing properties and was later developed into HpD and Photofrin to enhance its effectiveness in PDT (3). HpD and Photofrin are complexes of various porphyrin derivatives. The primary characteristic of these PSs is the presence of porphyrin structures, which absorb light, particularly in the red region of the electromagnetic spectrum, and interact with molecular oxygen to generate ROS that induce cell death (22). Despite their effectiveness in causing photo-damage to cells, these first-generation PSs have limitations. They have a relatively weak absorption at therapeutic wavelengths (>600 nm), limiting the depth of tissue penetration. Moreover, these agents also have low tumor selectivity, leading to damage to healthy tissues. Prolonged skin phototoxicity is another drawback, with patients becoming sensitive to light for several weeks after treatment (23).

### 2.2 Second-generation PSs

**Figure 1:**
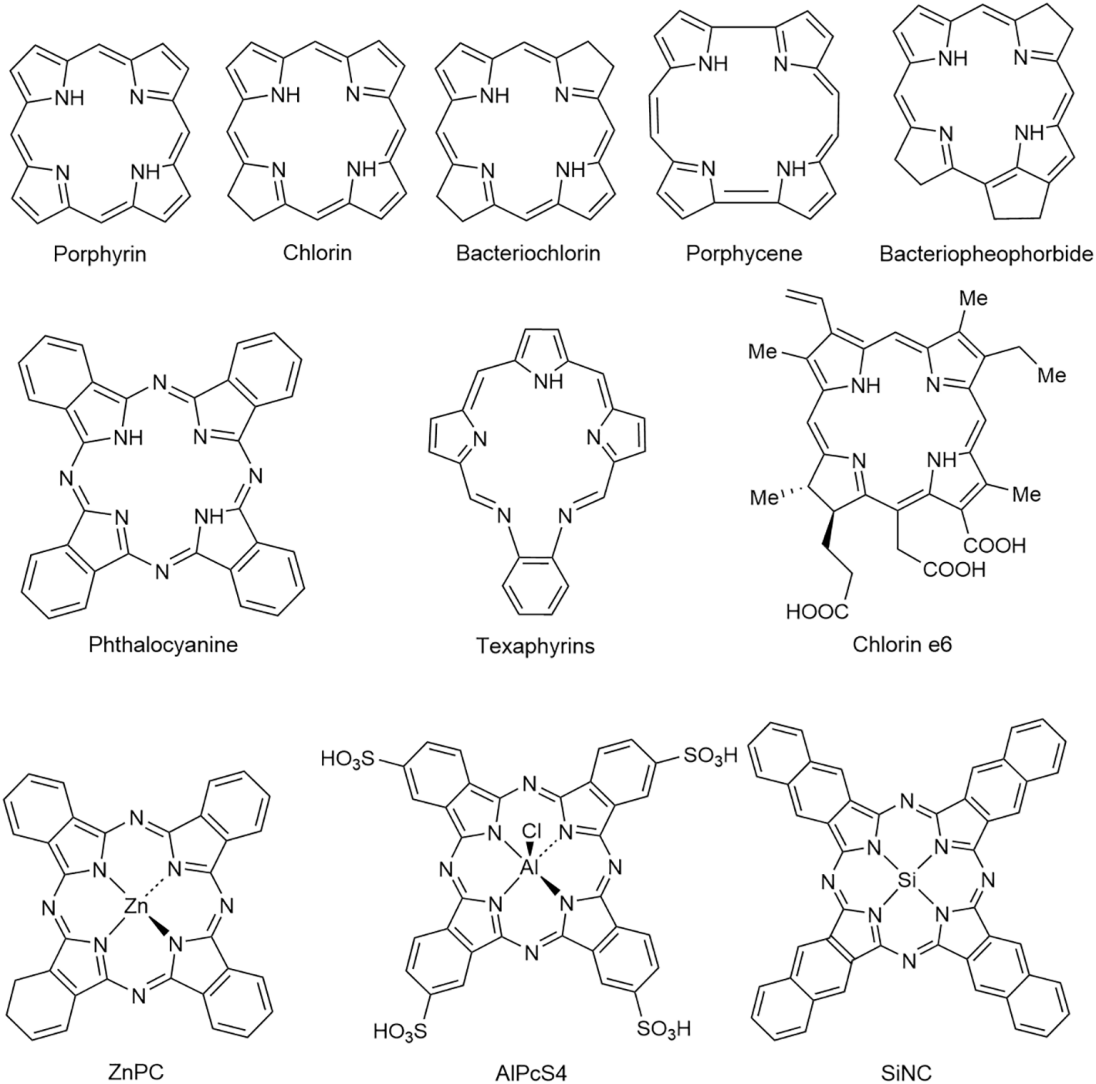
Structures of some second-generation PSs (24). Reproduced with permission from (24).

Due to the drawbacks of the first-generation PSs, more research was conducted on the second-generation PSs with strong ^1^O_2_ production and near-infrared (NIR) activation. Second-generation PSs include both porphyrinoid and non-porphyrinoid compounds (**Figure 1**) (24). The former consists of macrocyclic structures like porphyrin, chlorins, bacteriochlorins, phthalocyanines, pheophorbides, bacteriopheophorbides, Porphycene, and texaphyrins, whereas the latter comprises anthraquinones, phenothiazines, xanthenes, cyanines, and curcuminoids (24). Metalated derivatives such as aluminum phthalocyanine tetrasulfonate (AlPcS4), Si (IV)-naphthalocyanine (SiNC), zinc phthalocyanine (ZnPC), and tin ethyl etiopurpurin (SnET2) are also included, although metalation doesn’t consistently enhance photodynamic activity (25). Second-generation PSs are superior to HpD in terms of ^1^O_2_ quantum yields, tumor-to-normal tissue concentrations, and antitumor effects (26). They also offer practical advantages like shorter tissue accumulation time, enabling same-day treatment and making PDT more convenient for out-patient procedures. Furthermore, these PSs exhibit a reduced window of cutaneous photosensitivity (27). Several factors, including lipophilicity, the type and number of charged groups, charge-to-mass ratio, and the type and number of ring and core substituents, influence the properties of the PSs (28). Most of the second-generation PSs, particularly those with porphyrin ring structures, are hydrophobic, affecting their administration, biodistribution, and pharmacokinetic profile (11). Although hydrophobicity enables cellular penetration, extreme hydrophobicity could cause aggregation in aqueous solution, compromising its pharmacokinetics and photophysical properties. Moreover, it could restrict solubility in physiological solvents and body fluids, thus limiting clinical applications. Hence, a balance between hydrophilicity and lipophilicity is critical for a clinically successful PS. This balance can be achieved by introducing hydrophilic polar substituents in the lead PS structure to synthesize amphiphilic derivatives. Other modifications, such as oxidation, extension, or modification of the porphyrin ring system to carry a central ion, can also enhance photophysical and pharmacological properties (29).

### 2.3 Third-generation PSs

Present research primarily focuses on the development of third-generation PSs that are expected to enhance tumor specificity, minimize generalized photosensitivity, and be activated with longer-wavelength light (30). These improvements could be accomplished by modifying existing PSs with biological conjugates like peptides, antibodies, or antisense molecules for tumor-specific targeting, or by chemically conjugating or encapsulating PSs in efficient delivery vehicles or carriers (31–33). In essence, these third-generation PSs represent advancements over their second-generation counterparts in terms of improved delivery and targeting abilities. Specifically, they are designed to bind selectively to tumor cells or elements of the TME, consequently boosting tumor localization while reducing phototoxicity to healthy tissue.

## 3 Nanotechnology in PDT

Nanotechnology has emerged as a promising instrument for enhancing the delivery, efficacy, and safety of PSs in PDT (4). The application of nanoparticles as carriers of drugs offers several advantages, including improved solubility and stability, controlled drug release, and tumor-specific delivery of PSs (34). Additionally, targeted delivery of PSs and immunotherapeutic agents can be achieved using nanoparticles functionalized with specific ligands or antibodies, which can reduce off-target toxicity and enhance therapeutic efficacy (35). Nanoparticle-based systems have also been used to co-deliver PSs and immunotherapeutic agents, leading to a more potent and coordinated antitumor immune response (36, 37).

### 3.1 Organic nanoparticles

**Figure 2:**
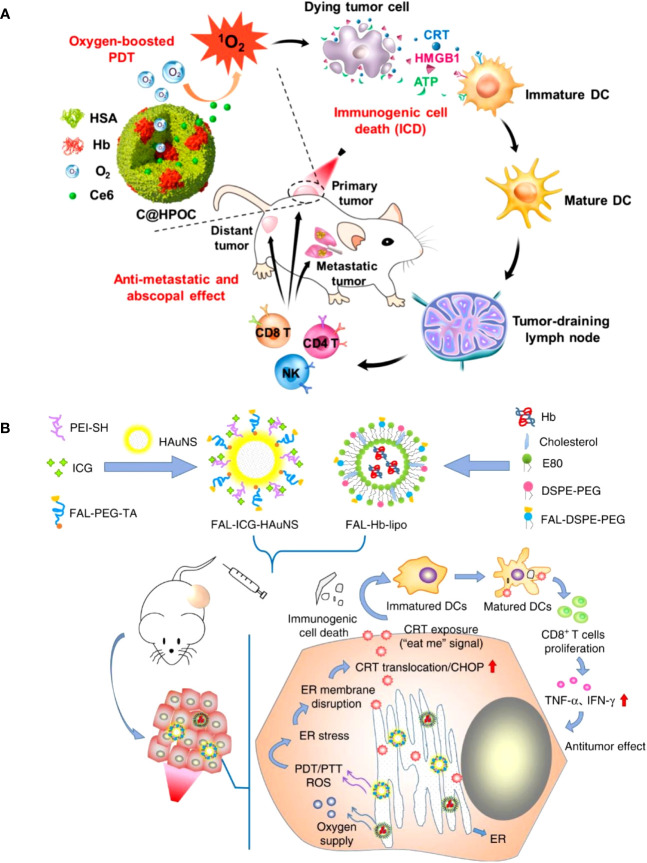
**(A)** Schematic depiction of oxygen-augmented immunogenic PDT with C@HPOC for eliciting the anti-metastatic and abscopal effect (40). Copyright 2018, American Chemical Society. **(B)** The antitumor mechanism of FAL-ICG-HAuNS plus FAL-Hb-lipo. Schematic illustration of enhanced immunogenic cancer cell death and anticancer effect induced by endoplasmic reticulum-targeting photothermal/photodynamic therapy (41). Copyright 2013, Nature.

Organic nanoparticles like micelles, liposomes, and dendrimers have been extensively investigated for PS delivery in PDT. Liposomes are spherical vesicles composed of phospholipid bilayers, which can encapsulate both hydrophilic and hydrophobic PSs (38). Micelles are self-assembled aggregates of amphiphilic molecules that can solubilize hydrophobic PSs within their hydrophobic core (39). Dendrimers are highly branched, tree-like polymers with a high degree of molecular uniformity and tunable surface functional groups, which can be used to conjugate PSs (39). Chen et al. developed a hybrid protein oxygen nanocarrier with chlorine e6 (Ce6) for oxygen self-sufficient PDT, which substantially decreased tumor hypoxia and improved the efficacy of PDT and the infiltration of CD8^+^ T cells at the cancer location (40). The increased PDT caused strong ICD and effectively inhibited primary tumors and suppressed pulmonary metastasis by stimulating more dendritic cells (DCs), NK cells, and cytotoxic T lymphocytes (CTLs) by increasing the release of damage-associated molecular patterns (DAMPs) (**Figure 2A**). A recent study improved the efficacy of PDT by using oxygen-laden hemoglobin (Hb) loaded auxiliary liposomes in conjunction with indocyanine green (ICG) modified gold nanospheres (41). Upon reaching the hypoxic tumor environment, the oxygen is rapidly released from the Hb, aiding the ICG in generating robust ROS and enhancing the immune response’s intensity (**Figure 2B**).

### 3.2 Inorganic nanoparticles

**Figure 3:**
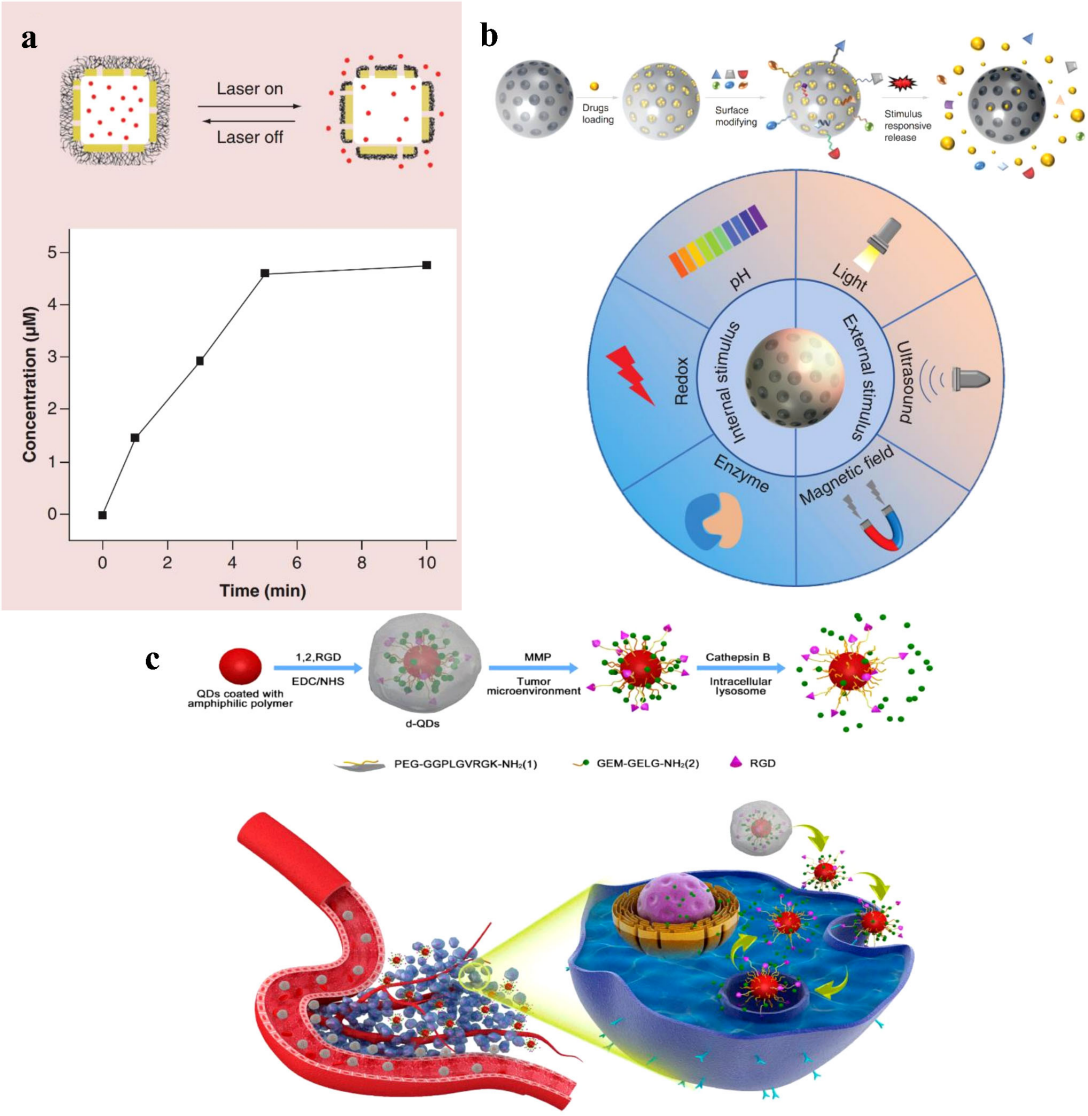
**(A)** A schematic illustration of controlled release enabled by a smart polymer, poly(N-isopropylacrylamide), coated on the surface of Au nanocages, and release profile of Dox from the system shown in above, as triggered by a NIR laser (42). Copyright 2016, Future Medicine Ltd. **(B)** Characteristics of controlled-release of mesoporous silica nanoparticles, and Characteristics of controlled-release of mesoporous silica nanoparticles (43). Copyright 2022, Future Medicine Ltd. **(C)** Schematic illustration of the nanovectors preparation protocol and their enzyme sensitive behavior, and Schematic illustration of the nanovectors delivering GeM to pancreatic cancer cells (44). Copyright 2017, American Chemical Society.

Inorganic nanoparticles, such as gold nanoparticles, silica nanoparticles, and quantum dots (**Figure 3**), have also been investigated for PS delivery (45, 46). Gold nanoparticles (AuNCs) can be easily functionalized with various ligands and PSs, allowing for targeted delivery and controlled release (47). Silica nanoparticles are highly biocompatible and can be modified with various functional groups to improve PS loading and release (43). Quantum dots, semiconductor nanoparticles with size-tunable optical properties, can act as both PSs and imaging agents in PDT (44). Manganese dioxide (MnO_2_) can stimulate the overproduction of hydrogen peroxide in tumor cells, resulting in the production of oxygen (48). Additionally, AuNCs act as intrinsic inorganic PSs that can induce ROS production, and the use of gold-based nanomedicines can enhance the effectiveness of other PSs in PDT due to the presence of a localized electric field (42, 49).

## 4 Advanced PSs for PDT

### 4.1 Nanoparticles improve PS properties

#### 4.1.1 Enhanced solubility and stability

**Figure 4:**
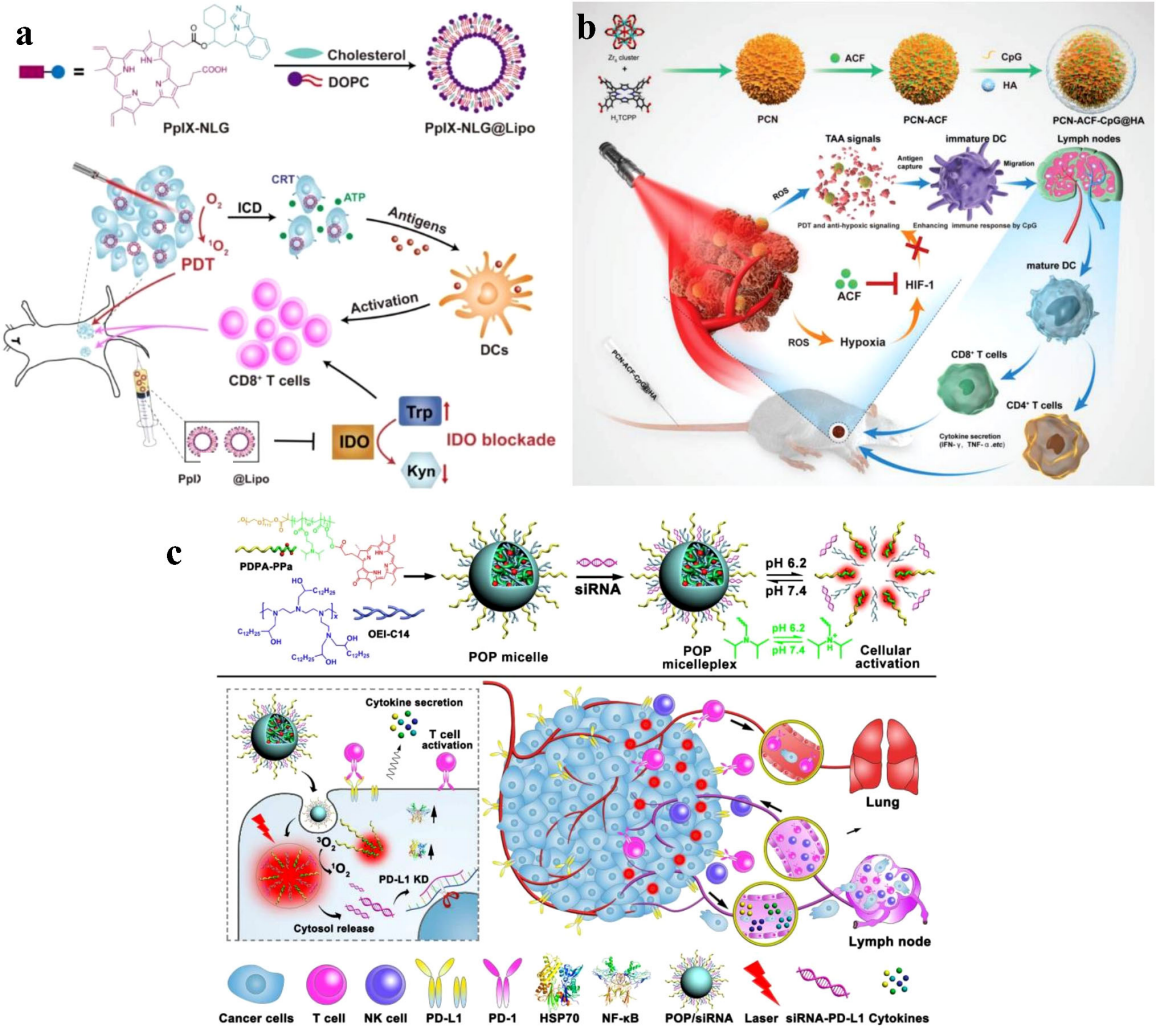
**(A)** Schematic illustration of PpIX-NLG@Lipo for combined PDT and IDO blockade (53). Copyright 2019, Ivyspring International. **(B)** Schematic illustration of the preparation procedure and the working principle of PCN-ACF-CpG@HA to integrate PDT, antihypoxic signaling, and CpG adjuvant as *in situ* tumor vaccine (54). Copyright 2019, John Wiley and Sons. **(C)** Schematic illustration of the acid-activatable micelleplexes for PD-L1 blockade-enhanced photodynamic cancer immunotherapy (55). Copyright 2016, American Chemical Society.

Many PSs exhibit poor solubility and stability in aqueous environments, which can limit their clinical application. Nanoparticles can improve the solubility and stability of PSs by encapsulating them within their hydrophobic core or conjugating them to the nanoparticle surface (50, 51). For example, liposomal formulations of the PS verteporfin have shown improved solubility and stability, resulting in enhanced PDT efficacy in preclinical studies (52). Huang et al. designed a dual-functional drug conjugate comprised of protoporphyrin IX and NLG919, a strong indoleamine-2,3-dioxygenase (IDO) inhibitor, to enhance the biocompatibility and tumor accumulation of the drug conjugate (PpIX-NLG@Lipo) (**Figure 4A**). *In vitro* and *in vivo* experiments demonstrated the strong ROS generation ability of PpIX-NLG@Lipo, which directly damages cancer cells through PDT. Concurrently, PpIX-NLG@Lipo induces ICD to stimulate the host immune system. Moreover, by interfering with IDO activity, PpIX-NLG@Lipo amplifies PDT-induced immune responses, resulting in an increased infiltration of CD8^+^ T lymphocytes at the tumor site and ultimately inhibiting both primary and distant tumors (53).

#### 4.1.2 Controlled release and distribution

Nanoparticles can be designed for controlled payload release, which improves the therapeutic index of PDT by maintaining optimal PS concentrations at the tumor site (56). By incorporating stimuli-responsive nanoparticles that release their payload in response to specific triggers, such as pH, temperature, or light, the spatiotemporal control of PS release can be further enhanced (57). This approach can potentially overcome the aggregation-caused quenching (ACQ) effect often seen in PDT, as well as increase the efficacy of the treatment by facilitating ROS diffusion (58, 59). Nanosized metal-organic Frameworks (nMOFs) incorporate PSs as structural units, producing adjustable and permeable structures that surmount the ACQ effect and permit significant PS loads (60, 61). These permeable structures not only efficiently disperse PSs but also promote ROS diffusion, thus improving the efficacy of PDT (58, 62). Cai et al. developed *in situ* tumor vaccines composed of MOF-based nanoparticles (PCNs), immunologic adjuvants (CpG), and hypoxia-inducible factor inhibitors (acriflavine) (54). To accomplish superior tumor targeting, the PCN-CpG-acriflavine surface was coated with hyaluronic acid (HA) to identify the increased CD44 receptor on the surfaces of tumor cells. In the research they conducted, self-assembled PCNs were made up of zirconium ions and H2TCPP, and the well-dispersed H2TCPP inside the framework counteracted the ACQ effect, resulting in equivalent PDT efficacy. In addition, the complementary impact of PDT-generated tumor-associated antigens (TAAs) and CpG resulted in robust immune responses (**Figure 4B**). Zhang et al. developed a hybrid cytomembrane (FM)-coated nMOF for photoactivatable immunotherapy of cancer to further enhance immune responses. FMs originate from DCs and tumor cells (63). The FMs not only exhibited exceptional tumor-targeting capacity because of their self-targeting properties toward homologous tumors but also facilitated tumor-specific immune responses related to the presence of extremely expressed tumor antigens. In addition, the DC-derived immunomodulatory molecules in FMs improved the presentation of antigen and encouraged antigen-specific T cell reactions.

Compared to normal tissues, solid tumors exhibit unique TME characteristics, including low pH, acute hypoxia, and increased glutathione (GSH) amounts (64). Designing intelligent, stimuli-responsive nanomedicines with TME-sensitive chemical linkages or components can therefore efficiently regulate cargo discharge. In addition to advanced nMOFs, TME-responsive nanoparticles can effectively surmount the ACQ by cleaving the TME-sensitive linkers, allowing for the quick dissolution of PSs at the tumor location and significantly boosting the generation of ROS (65). To facilitate PDT-based cancer immunotherapy, Wang et al. designed a pH-responsive, multipurpose nanoplatform (**Figure 4C**). This nanoplatform is composed of a pH-sensitive diblock copolymer (PDPA), pheophorbide A (PPa) attached to PDPA, and an inhibitor of the anti-programmed death-1 (PD-1)- anti-programmed death-ligand 1 (PD-L1) interaction (55). Due to the ACQ effect, the nanoplatform’s hydrophobic core encases PPa, reducing phototoxicity during blood circulation. The rapid micelle dissociation induced by PDPA’s “proton sponge effect” upon delivery to the mildly acidic TME restores PPa’s photoactivity. Consequently, irradiation-activated PPa generates ROS via PDT, inducing an immune response by stimulating the expression of NF-κB and HSP70.

### 4.2 Conjugation with targeting ligands

PSs can be conjugated with targeting ligands, such as antibodies, peptides, or small molecules, to enhance their tumor specificity and uptake (66, 67). Targeted delivery of PSs can be achieved using nanoparticles functionalized with specific ligands or antibodies that recognize receptors overexpressed on tumor cells or within the TME (68, 69). This strategy can improve the selectivity of PDT, reducing off-target toxicity and enhancing therapeutic efficacy. For example, folic acid-functionalized gold nanoparticles loaded with the PS chlorin e6 have demonstrated improved targeting and PDT efficacy in folate receptor-overexpressing cancer cells (70) A PS conjugated with an epidermal growth factor receptor (EGFR)-targeting peptide demonstrated improved PDT efficacy in EGFR-overexpressing cancer cells (71).

**Figure 5:**
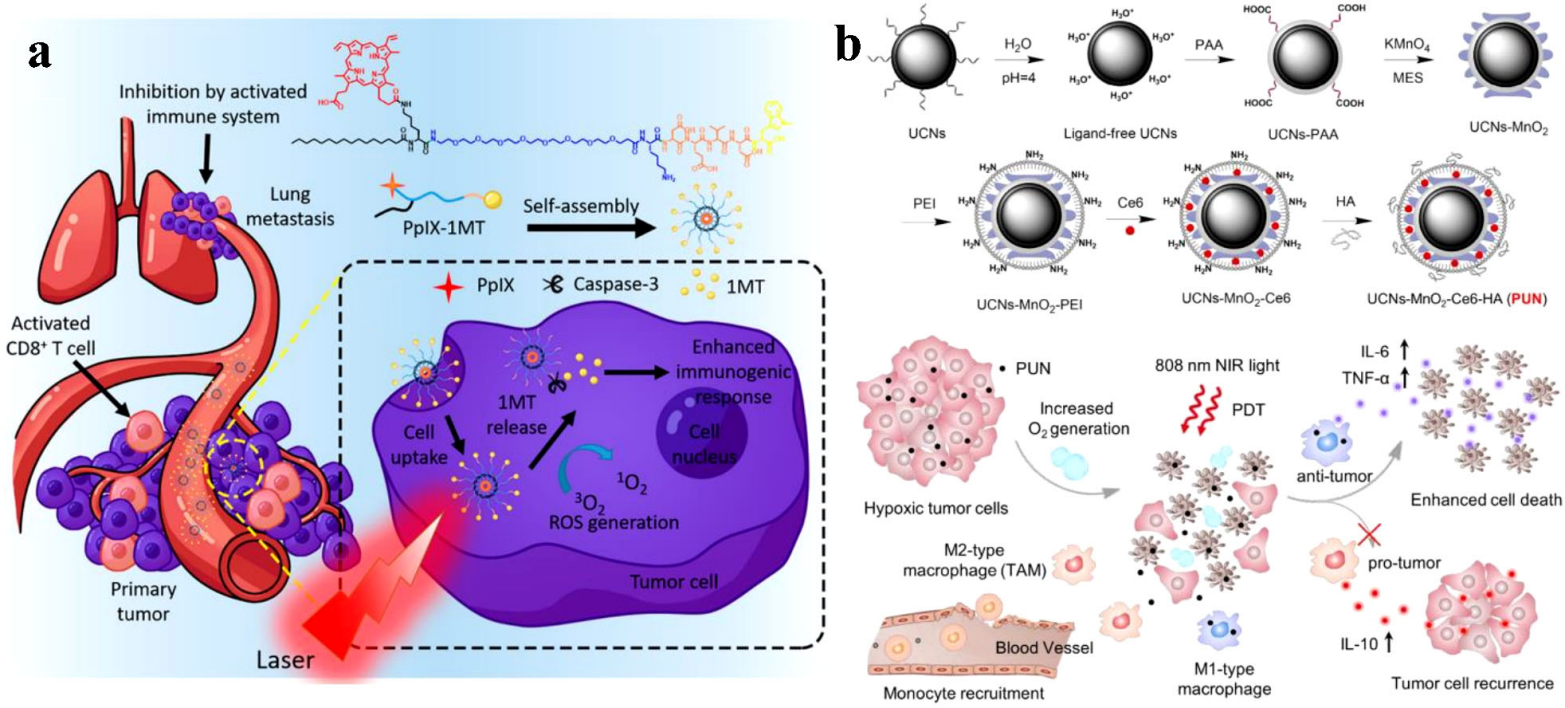
**(A)** PpIX-1MT chimeric peptide nanoparticles target tumor areas via the EPR effect, activate CD8+ T cells through cascade activations, and effectively inhibit primary tumors and lung metastasis while enabling *in situ* PDT to trigger apoptosis and caspase-3 production (75). Copyright 2018, American Chemical Society. **(B)** Illustration of NIR light-mediated PDT strategy for the enhanced cellular ablation in tumor microenvironment (76). Copyright 2018, American Chemical Society.

Due to the enhanced penetration and retention (EPR) effect that results from tumor vessel permeability, nanoparticles accumulate in tumor cells (72–74). Song et al. reported the development of PS-conjugated immune checkpoint inhibitor nanoparticles. As shown in **Figure 5A**, because of the EPR effect, these nanoparticles collect within tumors, Targeted delivery of PS and checkpoint inhibitor slowed tumor regrowth, prevented lung metastasis, and increased CD8^+^ T cells systemically (75). The hybrid nanoparticles that release PS and glucocorticoid-induced tumor necrosis factor receptor family-related protein, or poly (lactic-co-glycolic acid) (GITR-PLGA), utilize the immune activating role of PDT and GITR-PLGA-mediated suppression of immunosuppression to increase the amount of anti-tumor CD8^+^ T cells in the tumor (16).

### 4.3 Dual-function PSs and nanoparticles

Dual-function PSs have attracted considerable interest because of their multifunctional characteristics, that make them appropriate for imaging and treatment using PDT. This integrated method enables continuous tracking of the therapy process, enhancing the efficiency and precision of PDT (77). ICG is a well-known example of a dual-function PS. It is a water-soluble tricarbocyanine dye with widespread use in medical diagnostics for its fluorescence imaging capabilities (78). In recent years, researchers have also discovered its potential for PDT applications. ICG has shown efficacy in both *in vitro* and *in vivo* experiments, showing its potential for imaging and treating malignant tumors simultaneously (79). Porphysomes, another example of dual-function PSs, are self-assembled porphyrin-lipid nanoparticles. These unique structures exhibit excellent photoacoustic imaging and PDT properties. Porphysomes have been shown to generate a strong photoacoustic signal in response to laser irradiation, allowing for high-resolution imaging of targeted tissues. Moreover, porphysomes can generate cytotoxic ROS upon light activation, leading to targeted destruction of cancer cells (80).

### 4.4 Upconversion nanoparticles for deep-tissue activation

The restricted absorption of light into tissues limits treatment to surface tumors or requires invasive procedures for deeper tumors (27, 81). To overcome this limitation, researchers have explored strategies such as the use of upconversion nanoparticles (UCNPs), which convert NIR light to higher-energy light that is visible, allowing deeper tissue penetration without damaging surrounding tissues (82, 83).

UCNPs are nanoscale materials that convert low-energy light to high-energy light by sequentially exciting multiple photons via an anti-Stokes emission process. Compared with downconverted nanoparticles, UCNs can absorb NIR light and have a relatively high depth of tissue penetration, while the light can be converted into strong ultraviolet or visible light, enabling the activation of PSs in deep tissues (84). When coupled with PSs, UCNPs can extend the tissue penetration depth of PDT, improving its efficacy in the treatment of deep-seated tumors (85–89).

UCN-based PDTs have been extensively studied for tumor therapy due to their ability to improve tissue penetration depth. A study by Ai et al. utilized this feature by constructing Ce6-loaded UCNs combined with HA and MnO_2_ nanosheets to enhance NIR light-mediated PDT (**Figure 5B**). By converting 808 nm light excitation into 655 nm emissions, the Ce6-loaded UCNs efficiently generated sufficient ^1^O_2_ to induce deep-tissue cellular ablation. Additionally, the surface-anchored HA inhibited tumor recurrence post-PDT treatment by producing M1-type macrophages instead of M2-type macrophages (76).

## 5 Combining PDT and immunotherapy

### 5.1 Immunomodulatory effects of PDT

**Figure 6:**
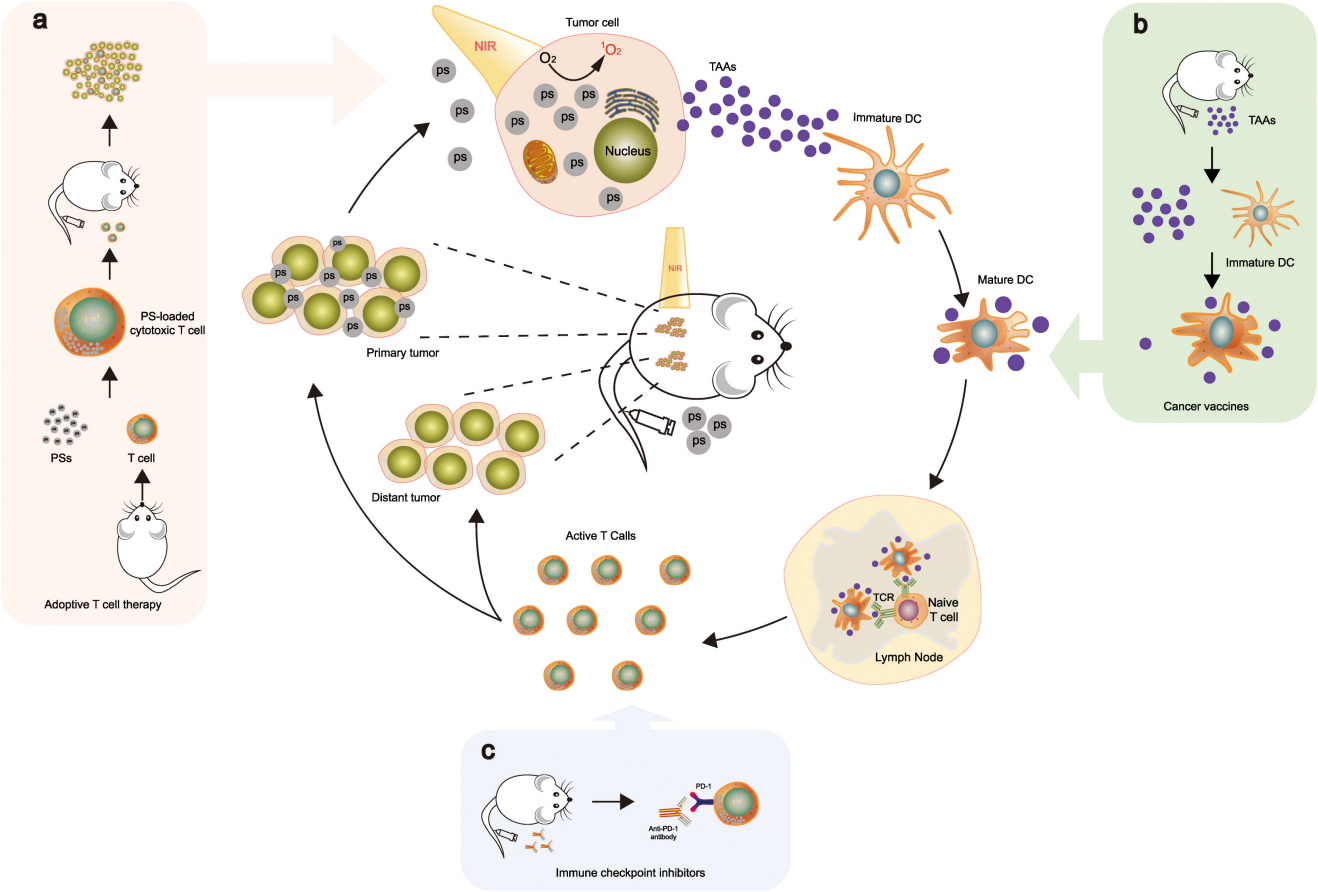
PDT triggers systemic antitumor immunity. This combination stimulates ICD and fosters an inflammatory environment at the primary tumor location, thereby releasing TAAs. These TAAs are then processed and introduced by dendritic cells to naive T cells, facilitating the growth and multiplication of tumor-specific effector T cells in lymphoid organs. The tumor-specific effector T cells infiltrate not only in primary but also in distant tumors. When combined with Adoptive T cell therapy, Cancer vaccine, and Immune checkpoint inhibitor, the mechanisms are as follows: **(A)** Ps is loaded into cytotoxic T cells; upon intravenous injection, they accumulate in the tumor tissues (92). **(B)** TAAs are utilized as a vaccine to enhance tumor-associated immune responses by dendritic cells presenting antigens to T cells (93). **(C)** Integration with PD-1 checkpoint blockade boosts the formation and infiltration of tumor-specific effector T cells in primary and distant tumors (94). The mechanisms **(A–C)** illustrate part of the drug mechanism of this therapy. Reproduced with permission from (92–94).

PDT has been demonstrated to elicit ICD, increasing immune responses, which stimulates the immune system against dying cancer cells (90, 91). When combined with Adoptive T cell therapy, Cancer vaccine, and Immune checkpoint inhibitor, the ideal mechanism is currently speculated as **Figure 6** (92–94). ICD promotes the release of DAMPs and TAAs, which act as danger signals to activate antigen-presenting cells (APCs) and initiate an immune response (95). PDT can activate APCs, such as DCs, through the release of DAMPs and the upregulation of costimulatory molecules. When immature DCs migrate from peripheral organs to neighboring draining lymph nodes, they gather antigens from the surrounding fluid, convert them into peptides, and then present the peptide major histocompatibility complex (MHC) at T cell receptors (TCR) triggering T cell activation, thereby priming and activating tumor-specific CTLs (96, 97). PDT has been reported to enhance the infiltration of T cells into the TME by modulating the expression of chemokines and adhesion molecules, thereby promoting the attraction of CTLs along with additional immune cells to the tumor location (98).

### 5.2 Types of immunotherapy used in combination with PDT

#### 5.2.1 Immune checkpoint inhibitors

In preclinical investigations, the combination of PDT with immune checkpoint inhibitors such as anti-cytotoxic T-lymphocyte-associated protein 4 (CTLA4), anti-PD-1, anti-PD-L1 antibodies and IDO inhibitor demonstrated synergistic effects (94, 99). This review synthesizes recent investigations into the utilization of nanomaterials in the combination of PDT with immune checkpoint inhibitors (**Table 1**). The combination boosts the immune system’s response to tumors and can surmount the immunosuppressive TME (105, 106). Liu et al. developed an effective approach to eliminate metastatic malignancies by combining oxygen-boosted PDT with checkpoint blockade therapy (107). Using a light-activated hydrogel with Ce6-modified CAT, biodegradable polymer, and an immune adjuvant, which induced strong ICD and regulated macrophage polarization, leading to a significantly enhanced immune response. This was achieved by raising the intratumoral CD8^+^ T cell accumulation. Wang et al. created a sequential delivery strategy to enhance therapeutic efficacy. They initially assembled a biocompatible polymer nanoparticle system by combining dextran-modified HAase (DEX-HAase) with pH-responsive linkers. Due to the second infusion of Ce6@liposome and irradiation, the remodeled TME enhanced the efficacy of PDT after the initial infusion of DEX-HAase nanoparticles. Notably, the intensified PDT induced a robust immune response, which increased by combining it with PD-L1 blockade therapy after the third anti-PD-L1 administration. This sequential infusion of therapeutic agents facilitated synergistic PDT-immunotherapy, effectively suppressing the growth of both primary and distant tumors (102).

**Table 1:** Summary of nanomaterials in the combination of PDT with immune checkpoint.

Nanomaterial	PS drug	Nanotechn-ology	Immuno-therapy targeting	Experim-ent	Tumor	Anti-meta-static	Imm-une-mem-ory	Ref.
NCP@pyrolipid	pyrolipid	nanoscale coordination polymer	PD-L1	vitro+vivo	colon	√	–	(36)
IDOi@TBC-Hf	TBC-Hf	nMOF	IDO(NLG919)	vivo	colon	√	–	(100)
DSAB-HK	phthalocyanine	dye-labeled probe	PD-1	vitro+vivo	breast	√	–	(94)
ZnP@pyro	pyrolipid	ZnP@pyro	PD-L1	vitro+vivo	breast	√	–	(45)
H-MnO2-PEG/C&D	Ce6	H-MnO2	PD-L1	vivo	breast	√	–	(46)
UCNP-Ce6-R837	Ce6	UCNPs	CTLA-4	vitro+vivo	colon	√	√	(83)
PpIX-1MT	PpIX	DEVD	IDO(1MT)	vitro+vivo	colon	√	√	(75)
PpIX-NLG@Lipo	protoporphyrin IX	liposome	IDO(NLG919)	vitro+vivo	breast	√	–	(53)
BMS-202 NPs	Ce6	reprecipitation	PD-1/PD-L1(BMS-202)	vivo	breast	√	√	(99)
Chloringlobulin	Ce6	Immunoglobu-lin G	PD-L1/CTLA-4	vitro+vivo	colon	√	√	(101)
IRD-αCD276/Fab	IRDye700	Fab preparation kit	PD-L1	vitro+vivo	breast	√	–	(99)
M-MONs@Ce6	Ce6	organosilica	CTLA-4	vitro+vivo	breast	√	–	(51)
Ce6@liposome-based	Ce6	liposome、DEX-Haase	PD-L1	vitro+vivo	breast	√	–	(102)
FIC	Ce6	–	PD-L1	vitro+vivo	Melan-oma/ colon	√	–	(103)
HS201-PDT	HS201	heat shock protein 90	PD-L1	vitro+vivo	breast	√	–	(104)

Furthermore, nMOFs can efficiently load immune checkpoint inhibitors into their open channels due to their high co-loading capacity. For example, Lin et al. constructed H4TBC-based nMOFs to encapsulate IDO inhibitors within their highly porous structure, inducing systemic antitumor immunity (100). Their results showed that H4TBC-based nMOFs provided both PDT and ICD, and when combined with checkpoint blockade therapy using an IDO inhibitor, enhanced T cell infiltration in the TME. In another study, Zhang et al. developed benzoporphyrin-based nMOFs composed of a Zr6 cluster and PS (TBP) in combination with αPD-1 to inhibit tumor metastasis (108). The TBP effectively avoided the ACQ effect due to its good dispersion in the nMOF structure, thereby facilitating PDT. Based on these advantages, TBP-mediated PDT induced a strong ICD to recruit tumor-infiltrating CTLs. Importantly, the combined application of αPD-1 restored the activity of CTLs suppressed in the immunosuppressive TME. In another study, Lin et al. constructed a cationic nMOF (W-TBP) highly loaded with anionic CpG through electrostatic interaction to enhance cancer immunotherapy (109). The combination of αPD-1 with antigen availability significantly improved the penetration and stimulation of CTLs in bilateral tumors, resulting in potent cancer immunotherapy.

#### 5.2.2 Cancer vaccines and adoptive T cell therapy

Combining PDT with cancer vaccines, which aim to motivate the immune system to identify and target tumor antigens, is possible. In preclinical models, the combination of PDT and cancer vaccines has demonstrated improved antitumor efficacy and immune memory (12, 110–113). **Table 2** summarizes the representative results of the current combination of PDT with cancer vaccines. In a mouse model, one study examined the combination of PDT and TLR5 agonist flagellin-adjuvanted tumor-specific peptide vaccination (FlaB-Vax) for increased PD-1 blockade-mediated melanoma inhibition. Results show the combination therapy significantly increased tumor-infiltrating effector memory CD8^+^ T cells and systemic IFNγ secretion, improving the therapeutic benefits of PD-1-targeting checkpoint inhibitor therapy for malignant melanoma (93).

**Table 2:** Summary of nanomaterials in the combination of PDT with cancer vaccine.

Nanomate-rial	Vaccine platform type	Autol-ogous or alloge-neic	Antig-en	PS drug	Immune expression	Immune-memory	Ref
PC-Cell@gel	Cellbased (tumor cell)	Autol-ogous	–	PEI-CE6	TAAs, mDC, CD8^+^↑ Treg↓	√	(111)
PDT-based DCvaccine	Cellbased (DC)	Autol-ogous	–	OR141	TAAs, CD8^+^, CD4^+^, IFN-γ↑	√	(112)
ALA-PDT-DC	Cellbased (DC)	Autol-ogous	–	ALA	TAAs, IFN-γ, TNF-α, CTL, mDC↑ IL-10↓	√	(113)
Lipo-PhA	Protein	Allog-eneic	FlaB-Vax	Lipo-PhA	TAAs, CTL, IFN γ↑	√	(93)
nMOF	DNA vaccine	Allog-eneic	CpG	H2TCPP	TAAs, IFN-γ, TNF-α, CTL, mDC↑	√	(54)
SPNI	Protein	Allog-eneic	TLR7	Semico-nducting polymer	TAAs, CD8^+^, CD4^+^, IFN-γ↑	√	(97)

(↑, upregulation; ↓, downregulation).

Adoptive T cell therapy (ACT) involves the diversion of ex vivo expanded or genetically engineered T cells into a patient to target cancer cells. Combining PDT with ACT has shown potential for increasing T cell infiltration and activation in the TME, leading to improved antitumor responses (114). Blaudszun et al. introduced a combined photodynamic and cancer immunotherapy strategy involving the adoptive transfer of PS-loaded cytotoxic T cells (PS-OT-1 cells). They loaded OT-1 cells with temoporfin, a clinically useful porphyrin derivative, to create PS-OT-1 cells. Remarkably, under visible light irradiation in culture, PS-OT-1 cells produced a substantial quantity of ROS in an efficient manner. In addition, the combination of PDT and ACT with PS-OT-1 cells resulted in substantially increased cytotoxicity in comparison to ACT alone with unloaded OT-1 cells (92).

### 5.3 Preclinical and clinical studies on combined treatment strategies

Several preclinical studies have demonstrated the potential of combining PDT with immunotherapy, resulting in improved antitumor effects and long-lasting immune responses (36, 101). In a mouse model of melanoma, the combination of PDT and anti-PD-1 therapy led to enhanced tumor regression and improved survival rates (99, 115, 116). Another study showed that combining PDT with a cancer vaccine increased antitumor immunity and prevented tumor recurrence in a mouse model of colon cancer (117). Bao et al. targeted IRDye700 to subcutaneous murine 4T1 tumors using a Fab portion of an antibody that attaches to CD276, an antigen specifically expressed on tumor cells. While targeted NIR therapy reduced tumor regeneration, PD-L1 expression on tumor cells increased significantly. By combining CD276-targeted NIR-PIT and anti-PD-L1 therapy, they were able to prevent subcutaneous tumor regrowth and lung metastasis (69). Hao et al. developed a combined therapy approach using PDT and immune checkpoint blockade to optimize tumor control. They incorporated a 25% thermosensitive polymer 407 hydrogel as a co-delivery platform for this treatment strategy. NIR PDT at 808 nm irradiation, along with CTLA4 and PD-L1 checkpoint blockade, suppressed solid tumor growth and extended the survival rate of colorectal tumor-bearing mice. This was achieved by eliciting a range of immune responses, including macrophage and DCs phagocytosis of tumor debris, acute inflammation induction, leukocyte infiltration, as well as maturation and activation of DCs (20). In the study by Santos and colleagues, the use of PDT with redaporfin successfully eradicated all observable tumor tissue. Subsequently, the application of an immune checkpoint inhibitor contributed to a sustained complete response in the clinical setting. This particular case exemplifies the potential effectiveness of such a combined therapeutic approach, laying the foundation for the development of a novel treatment strategy (118).

## 6 Challenges and future directions

### 6.1 Overcoming side effects

Potential side effects of PDT, including skin photosensitivity, pain, and edema, stem from prolonged presence and systemic distribution of PSs (119). Addressing these concerns, nanotechnology provides targeted PS delivery, reducing off-target phototoxicity. Biodegradable CaCO3/MnO2-based nanocarriers have been successfully used to transport PSs, demonstrating significant advancements over potentially toxic gold-based nanomedicines (120). These nanoparticles, engineered to accumulate in tumors through the enhanced EPR effect, limit PS exposure in healthy tissues (103). Surface modifications with tumor-specific ligands further enhance nanoparticle specificity, minimizing non-target skin photosensitivity (4, 104). Some nanoparticles also provide controlled PS release in response to stimuli such as pH, temperature, or enzymes, localizing PS activation to the treatment area (121).

### 6.2 Personalized cancer treatment strategies

An emerging trend in cancer treatment is the development of personalized medicine, which tailors treatments to the distinctive features of a patient’s tumor (122, 123). The combination of PDT and immunotherapy allows for a more tailored approach by considering factors such as TME, immune profile, and response to treatment (94, 124). Identifying predictive biomarkers and developing treatment algorithms based on individual patient profiles will be crucial for improving patient outcomes (124).

### 6.3 Translational research for clinical implementation

Translating preclinical research findings into clinical practice remains a challenge for the field. Issues such as regulatory approval, manufacturing, and standardization of treatment protocols need to be addressed to ensure the successful clinical translation of nanotechnology-enabled PDT and immunotherapy (18, 125). Collaborative efforts between researchers, clinicians, and regulatory agencies are necessary to accelerate the development and approval of these innovative therapies (126, 127).

In conclusion, the exciting interplay of nanotechnology-enabled PDT, advanced PSs, and immunotherapy heralds a promising new era in cancer treatment. The potential to overcome limitations such as tissue penetration and side effects, coupled with the opportunity to develop personalized cancer treatment strategies, paves the way for successful clinical translation. At the heart of this revolutionary approach lies the convergence of several disciplines. Material science, molecular biology, and biomedical engineering come together to optimize nanomaterials for improved delivery and activation of PSs, enhancing biocompatibility and maximizing therapeutic payloads. Moreover, an in-depth understanding of the TME is vital to design nanoparticles capable of navigating and penetrating these complex terrains effectively. Simultaneously, insights from the intersection of immunology and oncology spotlight the potential of leveraging PDT to stimulate potent immune responses against tumors. This accentuates the exciting prospects of integrating PDT and immunotherapy for comprehensive cancer treatment. Furthermore, the introduction of machine learning and computational modeling can advance nanotherapeutic design by predicting biological interactions, leading to more effective and targeted therapies. The translation of these advancements from lab to clinic necessitates meticulous testing and a thorough understanding of regulatory pathways. This underlines the significance of collaborative efforts between researchers, clinicians, and regulatory agencies. Ultimately, the future of PDT and nanotechnology, through this multi-disciplinary collaboration, holds immense potential in revolutionizing cancer therapy, improving patient outcomes, and providing benefits to cancer patients worldwide.

## Author contributions

JJ and XW made equal contributions to this manuscript. JJ and XW prepared and wrote the manuscript. JY and GL revised the manuscript for clarity, grammar, and style. HZ and WH commented on previous versions of the manuscript. ZY and DW provided critical feedback on the manuscript. JT designed and revised the manuscript. All authors discussed the relevant literature and composed the sections of the review article. All authors contributed to the article and approved the submitted version.
